# Causal associations of cognition, intelligence, education, health and lifestyle factors with cervical spondylosis: a mendelian randomization study

**DOI:** 10.3389/fgene.2024.1297213

**Published:** 2024-04-25

**Authors:** Zhenxiao Ren, Xing Cheng, Jinghui Xu, Tianzuo Niu, Houqing Long

**Affiliations:** ^1^ Department of Spine Surgery, Orthopaedic, Shenzhen People’s Hospital (The Second Clinical Medical College, Jinan University/The First Affiliated Hospital, Southern University of Science and Technology), Shenzhen, Guangdong Province, China; ^2^ Guangdong Provincial Key Laboratory of Orthopaedics and Traumatology/Department of Spine Surgery, The First Affiliated Hospital, Sun Yat-sen University, Guangzhou, Guangdong Province, China; ^3^ Department of Spine Surgery, Orthopedics Center of Guangdong Provincial People’s Hospital (Guangdong Academy of Medical Sciences), Southern Medical University, Guangzhou, Guangdong Province, China

**Keywords:** cervical spondylosis, mendelian randomization, mediation analyses, education, sex hormone binding globulin

## Abstract

**Background:** Education, cognition, and intelligence are phenotypically and genetically related. Education has been shown to have a protective effect on the risk of developing cervical spondylosis. However, it is unclear whether cognition and intelligence have independent causal effects on cervical spondylosis, and whether health and lifestyle factors influence this association.

**Methods:** We first assessed the independent effects of education, cognition, and intelligence on cervical spondylosis by two-sample Mendelian randomization and multivariable Mendelian randomization analysis, and evaluated 26 potential association mediators using two-step Mendelian randomization, and calculated the median proportion.

**Results:** The results showed that only education had an independent causal effect on cervical spondylosis, and had a protective effect on the risk of cervical spondylosis (β: 0.3395; se: 0.166; *p* < 0.05; OR:0.71; [95%CI: 0.481–0.943]. Of the 26 potential associated mediators, a factor was identified: SHBG (mediated proportion: 2.5%). Univariable Mendelian randomization results showed that the risk factors for cervical spondylosis were time spent watching TV (OR:1.96; [95%CI: 1.39–2.76]), smoking (OR:2.56; [95%CI: 1.061–1.486]), body mass index (OR:1.26; [95%CI: 1.124–1.418]), percentage of body fat (OR:1.32; [95%CI: 1.097–1.593]), major depression (OR:1.27; [95%CI: 1.017–1.587]) and sitting height (OR:1.15; [95%CI: 1.025–1.291]). Protective factors include computer using (OR:0.65; [95%CI: 0.418–0.995]), sex hormone binding globulin (OR:0.87; [95%CI: 0.7955–0.951]) and high-density lipoprotein (OR:0.90; [95%CI: 0.826–0.990]).

**Conclusion:** Our findings demonstrate the causal and independent effects of education on cervical spondylosis and suggest that lifestyle media may be a priority target for the prevention of cervical spondylosis due to low educational attainment.

## 1 Introduction

The process of cervical spondylosis is a natural aging process, which is mainly manifested by progressive degenerative changes in the components of the cervical spine such as the intervertebral disc, ligament flavum, lamina, facet joint, etc (Bernabeu-Sanz et al., 2020; Kuo and Tadi, 2023). Congenital spinal stenosis, severe spinal trauma, and certain sports are the causes of early onset or accelerated progression of cervical spondylosis (Kelly et al., 2012; Lu et al., 2017). This process is often associated with aging and occurs after the age of 50, with the most commonly affected segments C6-7, followed by C5-6. The most common symptom is neck pain, which has a lifetime morbidity of up to 86.8%, which, along with low back pain, is the main cause of years lived with disability (Hoy et al., 2010; Hurwitz et al., 2018; Kuo and Tadi, 2023). In recent years, studies have pointed out that factors such as smoking, obesity, exercise, lifestyle and cardiovascular disease may be associated with the risk of cervical spondylosis (Theodore, 2020; Sun et al., 2023). With socio-economic development, the health resources and lifestyles people receive can be influenced by cognition, intelligence, and education. In addition, cognition, intelligence, and education have been shown to be phenotypically and genetically associated (Wang et al., 2023), as well as for hypertension and some cardiometabolic mediators. Education has now been shown to protect against the risk of cervical spondylosis (Sun et al., 2023). However, it is unclear whether cognition and intelligence have independent causal effects on cervical spondylosis, and whether health and lifestyle factors influence this association.

As a causal reasoning method, Mendelian randomization (MR) identifies Genome-wide single-nucleotide polymorphisms (SNPs) by using genome-wide association studies (GWAS). SNPs were used as genetic tools to explore the causal relationship between exposure of interest and outcomes. MR avoids the impact of reverse causal, environmental, and behavioral factors that can occur in observational studies (Lawlor et al., 2008). Multivariable Mendelian randomization (MVMR) is an extension of MR that allows different genetic variants of different exposures with correlation to be placed in the same study to determine the independent effects of different exposures on outcomes and to adjust potential pleiotropy (Sanderson et al., 2019).

In this study, we explored the independent causal relationship between cognition, intelligence and education and cervical spondylosis by using MR. Besides, the role of different lifestyle and health factors in the pathogenesis of cervical spondylosis is of great concern to us. Understanding this topic can improve the understanding of the etiological risks of cervical spondylosis, thereby providing prevention and intervention strategies for cervical spondylosis.

## 2 Methods

### 2.1 Study design

**FIGURE 1:**
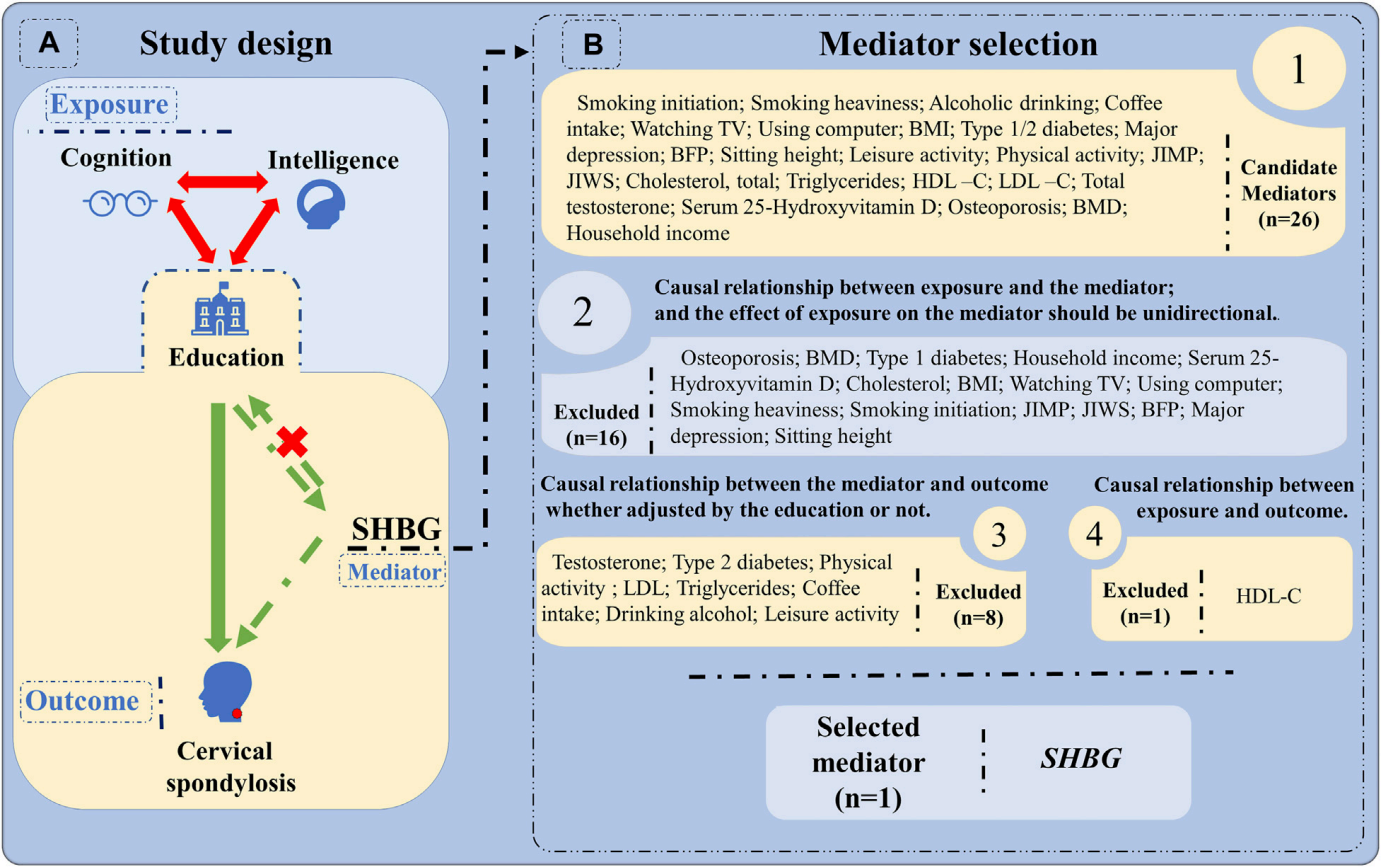
Flow chart of this article. **(A)** Study design: Firstly, the three exposure factors (education, cognition and intelligence) and cervical spondylosis were analyzed by UVMR and MVMR. Determine that education consistently has a causal relationship with cervical spondylosis, whether or not adjusted for other exposures. Secondly, determine the intermediary factors. **(B)** Mediator selection (Bernabeu-Sanz et al., 2020): There is a causal relationship between exposure and the mediator, and the effect of exposure on the mediator should be unidirectional (Kuo and Tadi, 2023). There is always a causal relationship between the mediator and the outcome, whether adjusted for the outcome or not (Kelly et al., 2012). There is always a causal relationship between exposure and outcome. SHBG: Sex hormone binding globulin; BMI: Body mass index; BFP: Body fat percentage; BMD: Bone mineral density; JIMP: Job involves heavy manual or physical work; JIWS: Job involves mainly walking or standing; HDL-C: high-density lipoprotein cholesterol; LDL-C: low-density lipoprotein cholesterol.

In this study, we first evaluated the causal relationship between cognition, intelligence, education and cervical spondylosis through univariable Mendelian randomization (UVMR) and MVMR. UVMR results showed that cognition, intelligence, education and cervical spondylosis were all causally related. MVMR results showed that only education had an independent causal relationship with cervical spondylosis and inter-regulated with intelligence and cognition. We then screened for two factors associated with cervical spondylosis and calculated the mediating effect using TWO-STEP-MR (Figure 1).

### 2.2 Data sources

In this study, the source of data for exposure, outcomes, and mediators was genome-wide association studies (GWASs). A total of 30 GWAS data were used for UVMR and MVMR testing (Table 1; Supplementary Table S5). All studies were limited to people of European ancestry to reduce bias due to population differences.

**TABLE 1:** The selected genome-wide association studies (GWAS) databases were shown in table. A total of 30 GWAS data were used and all studies were limited to people of European ancestry to reduce bias due to population differences. SHBG: Sex hormone binding globulin; BMI: Body mass index; BFP: Body fat percentage; BMD: Bone mineral density; JIMP: Job involves heavy manual or physical work; JIWS: Job involves mainly walking or standing; HDL-C: high-density lipoprotein cholesterol; LDL-C: low-density lipoprotein cholesterol.

Phenotype	Sample size	Number of SNPs	Population	Author	Publication time
Exposure					
Cognition	257,841	10,066,414	European	Lee et at	2018
Intelligence	269,867	9,276,181	European	Savage et al	2018
Education	766,345	10,101,242	European	Lee et al	2018
Outcome					
Cervical spondylosis	171956	16,380,237	European	NA	2021
Mediator					
SHHG	185,221	12,321,875	European	Rebecca Richmond et al	2020
Mediator(cx eluded)					
Smoking initiation	607,291	11,802,365	European	Liu Met al	2019
Smoking heaviness	249752	12003613	European	Liu M et al	2019
Alcoholic drinking	335,394	11,887,865	European	Liu, M et al	2019
Coffee intake	428,860	9,851,867	European	Ben Elsworth et al	2018
Watching TV	437,887	9,851,867	European	Ben Elsworth et al	2018
Using computer	360,895	9,851,867	European	Ben Elsworth et al	2018
BMI	454,884	9,851,867	European	Ben Elsworth et al	2018
Type 1 diabetes	189,113	16,380,008	European	NA	2021
Type 2 diabetes	215,834	16,380,440	European	NA	2021
Major depression	NA	NA	European	Howard DM et at	2019
BFP	331,117	10,894,596	European	Neale et al	2017
Sitting height	461,536	9,851,867	European	'Ben Elsworth et al	2018
Leisure activity	461,369	9,851,867	European	Ben Elsworth et al	2018
Physical activity	377,234	11,808,007	European	Klimentidis YC et al	2018
JIMP	263,615	9,851,867	European	Ben Elsworth et al	2018
JIWS	263,556	9,851,867	European	Ben Elsworth et al	2018
Cholesterol, total	94,595	2,418,562	European	Willer CJ et al	2013
Triglycerides	94,595	2,410,057	European	Willer CJ et al	2013
HDL —C	94,595	2418527	European	Willer CJ et al	2013
LDL —C	94,595	2,409,690	European	Willer CJ et al	2013
Total Testosterone	199,569	12,321,875	European	Rebecca Richmond et al	2020
Serum 25-Hydroxyvitamin D	417,580	8,401,108	European	Revez JA et al	2020
Osteoporosis	462,933	9,851,867	European	Ben Elsworth et al	2018
BMD	18,805	10,304,110	European	Medina-Gomez C et al	2018
Household income	397,751	9,851,867	European	Ben Elsworth et al	2018

#### 2.2.1 Exposure and outcome

The Intelligence Genetic instruments were selected from a GWAS analysis of 269,867 European individuals. Genetic instruments for education and cognition were selected from GWAS analyses involving 766,345/25,841 European individuals. For outcomes, we searched the Finngen Biobank for data on cervical spondylosis, and a GWAS analysis covering 171,956 European individuals was selected. First, to ensure that the genetic tools were strong, we extracted SNPs with relevant *p* values (*p* < 5 × 10^−8^) from the above GWAS datasets. Later, in order to avoid inaccurate results caused by the calculation of related SNP, linkage disequilibrium clumping (*R* ^2^ ≤ 0.001, window size = 10,000 kb) was performed for each MR Exposure. When *R* ^2^ > 0.001, SNPs with larger associated *p*-values will be deleted to ensure the accuracy of the results. When there are no specific SNPs in the exposure factor dataset, we use proxy SNPs by labeling LD (minimum LD Rsp value = 0.8; MAF threshold = 0.3).

#### 2.2.2 Mediators

Through the search of the existing literature, it is shown that smoking, obesity, exercise, lifestyle and cardiovascular disease may be associated with the incidence of cervical spondylosis (Theodore, 2020; Sun et al., 2023). Therefore, we chose smoking (smoking initiation, smoking heaviness); obesity (body mass index (BMI), body fat percentage (BFP)); leisure activity (moderate to vigorous physical activity levels); lifestyle (job involves heavy manual or physical work (JIMP), job involves mainly walking or standing (JIMS), time spent using computer, time spent watching television (TV), playing computer games, alcohol drinking, coffee intake, household income, sitting height); cardiovascular factors (total cholesterol, triglycerides, low-density lipoprotein cholesterol (LDL-C), high-density lipoprotein cholesterol (HDL-C)); In addition, we selected factors related to the body [diabetes (type 1/2 diabetes); sex hormones (sex hormone binding globulin (SHBG), testosterone, estrogen); Bone-related (osteoporosis, bone mineral density (BMD), serum 25-Hydroxy vitamin D); and major depression. We analyzed exposure (education), outcomes (cervical spondylosis) and mediators according to the following principles (Bernabeu-Sanz et al., 2020): There is a causal relationship between exposure and the mediator, and the effect of exposure on the mediator should be unidirectional (Kuo and Tadi, 2023). There is always a causal relationship between the mediator and the outcome, whether adjusted for the outcome or not (Kelly et al., 2012). There is always a causal relationship between exposure and outcome (Lu et al., 2017). The causal direction between exposure and mediation, between mediation and outcome, should be consistent. The mediating factors was SHBG.

### 2.3 Statistic analysis

All analyses MR satisfy three key assumptions (Bernabeu-Sanz et al., 2020): the genetic variants must be strongly associated with exposure and must be associated with at least one exposure in the MVMR (Kuo and Tadi, 2023); Genetic variants was not associated with any confounding factors associated with expose and outcome (Kelly et al., 2012); Genetic variants does not affect the results, unless the effect on cervical spondylosis is after each exposure (Hemani et al., 2018a). Inverse variance weighted (IVW): IVW is the most important MR analysis and forces the linear regression intercept to be set to 0. In IVW, it is assumed that the individual genetic variants are independent of each other. When there is a plurality of potentials, results can be skewed (Hemani et al., 2018b). MR-Egger: This method does not enforce a linear regression intercept of 0, so the intercept can be used to assess whether genetic variants has directional pleiotropism for the results (Bowden et al., 2017). Furthermore, the MR-Egger method is less accurate than the weighted median because it may be affected by peripheral genetic variation. Weighted median: When >50% of the information is useful for the analysis of valid instrumental variables, this method provides estimates that are compatible with the final effect. MR-PRESSO: It is currently widely used to test horizontal pleiotropy (Verbanck et al., 2018). We also applied the Q′ heterogeneity statistic to assess heterogeneity between instruments and condition F statistic to test instrument validity (Bowden et al., 2019). When F > 10, we can consider the instrument validity to be high.

UVMR: We used UVMR to estimate the effects of cognition, intelligence, and education on cervical spondylosis, respectively. For UVMR, we determined IVW as the main basis for MR Analysis. The weighted median and MR-Egger based regression method are combined to ensure the accuracy of the conclusion. In addition, horizontal pleiotropy was tested using the MR-PRESSO method. MVMR: It is an extension of MR by combining multiple potentially relevant exposure-related genetic variants to calculate the independent effect of each exposure on the outcome (Sanderson, 2021). For MVMR, we used IVW results as the main basis for MVMR, and used Q’ heterogeneity statistics to assess inter-tool heterogeneity.

Mediation Analyses: To further determine the overall effect rate of education on cervical spondylosis, we performed mediation analysis using the two-step-MR approach. First, we estimate the causal effect of exposure factors on the mediator (β1) using UVMR. Then, the MVMR method was used to estimate the causal effect of mediator on cervical spondylosis (β2). The indirect effect is obtained by multiplying (β1Xβ2) by the total effect. In this study, the association between all exposures and outcome phenotypes was statistically significant at *p* < 0.05. All of our analyses were performed using the “TwoSampleMR”, “MRPRESSO”, “MRPracticals” R packages.

## 3 Results

### 3.1 Casual associations of cognition, intelligence, education with cervical spondylosis

We carried out UVMR analysis on the causal relationship between cognition, intelligence, education and cervical spondylosis respectively: longer education time (β: 0.604; se: 0.094; *p* < 0.05; OR:0.54; [95%CI: 0.454–0.657]), higher intelligence (β: 0.43; se: 0.10; *p* < 0.05; OR:0.65; [95%CI: 0.533–0.796]), better cognition (β: 0.423; se: 0.090; *p* < 0.05; OR:0.66; [95%CI: 0.549–0.783]) had protective effects on the incidence of cervical spondylosis.

**FIGURE 2:**
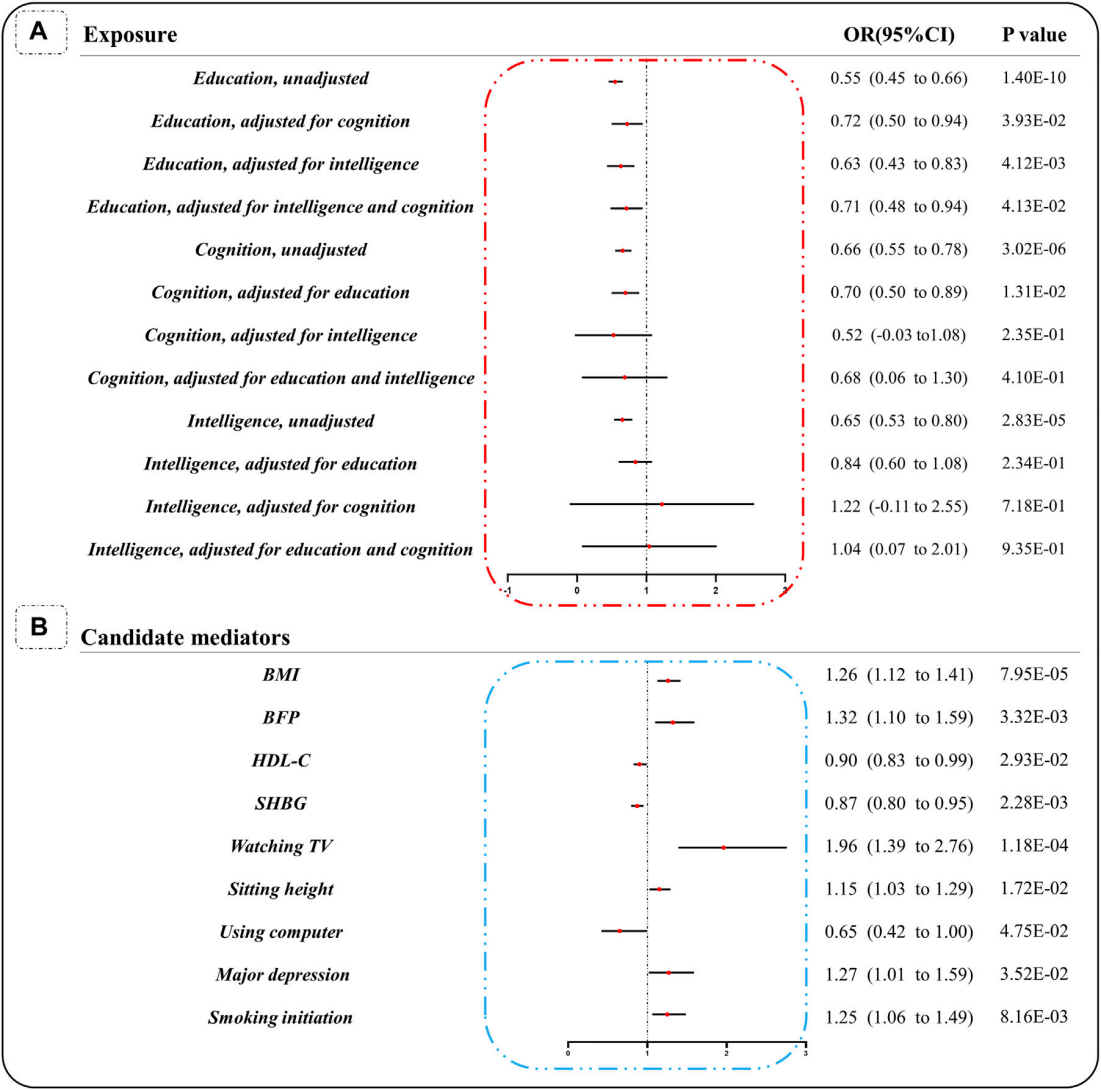
MR estimates of the causal associations of exposures, candidate mediators with cervical spondylosis. **(A)** Causal relationship between the exposures (education, cognition and intelligence) and cervical spondylosis, whether or not adjusted for other exposures. **(B)** UVMR analysis was performed on 26 candidate mediators and cervical spondylosis, and nine mediators were identified as having causal relationship with cervical spondylosis. OR indicates odds ratio and plots (bars) represent OR (95% CI). SHBG: Sex hormone binding globulin; BMI: Body mass index; BFP: Body fat percentage. HDL-C: high-density lipoprotein cholesterol.

MVMR results showed that there was a statistically significant causal relationship between education and cervical spondylosis, although it was accompanied by intelligence (β: 0.464; se: 0.161; *p* < 0.05; OR:0.63; [95%CI: 0.431–0.827]), cognition (β: 0.326; se: 0.158; *p* < 0.05; OR:0.72; [95%CI: 0.498–0.945]) or both (β: 0.3395; se: 0.166; *p* < 0.05; OR:0.71; [95%CI: 0.481–0.943]). After adjustment for education and cognition, the causal relationship between intelligence and cervical spondylosis was no longer statistically significant. The causal relationship between cognition and cervical spondylosis was statistically significant when only adjusted for education (β: 0.362; se: 0.145; *p* < 0.05; OR:0.70; [95%CI: 0.498 to 0.894]), which was not statistically significant when adjusted for intelligence or both (Figure 2).

### 3.2 Influence of candidate mediating factors on cervical spondylosis

Through MR analysis, we selected 26 candidate mediators and performed causal analysis of education with mediators, mediators with cervical spondylosis. Then, nine media were identified. Smoking was associated with cervical spondylosis (Smoking initiation had an adverse effect on cervical spondylosis (β: 0.228; se: 0.086; *p* < 0.05; OR:1.25; [95%CI: 1.061–1.486]), there was no causal relationship between smoking heaviness and cervical spondylosis (β: 0.072; se: 0.068; *p* > 0.05; OR:1.07; [95%CI: 0.940–1.228]). Time spent using computer was a protective factor for cervical spondylosis (β: 0.438; se: 0.221; *p* < 0.05; OR:0.65; [95%CI: 0.418–0.995]) and time spent watching TV was a risk factor for cervical spondylosis (β:0.672; se: 0.174; *p* < 0.05; OR:1.96; [95%CI: 1.39–2.76]). In addition, BMI (β:0.233; se: 0.059; *p* < 0.05; OR:1.26; [95%CI: 1.124–1.418]), BFP (β:0.279; se: 0.095; *p* < 0.05; OR:1.32; [95%CI: 1.097–1.593]), major depression (β:0.239; se: 0.113; *p* < 0.05; OR:1.27; [95%CI: 1.017–1.587]) and sitting height (β:0.140; Se: 0.059; *p* < 0.05; OR:1.15; [95%CI: 1.025–1.291]) were risk factors for cervical spondylosis. And SHBG (β: 0.140; se: 0.046; *p* < 0.05; OR:0.87; [95%CI: 0.795–0.951]) and HDL-C (β: 0.101; se: 0.046; *p* < 0.05; OR:0.90; [95%CI: 0.826–0.990]) were protective factors of cervical spondylosis (Figure 2). Otherwise, no causal relationship was found between exercise (leisure activities, physical activity); lifestyle (JIMP, JIMS, alcohol drinking, coffee intake, household income); cardiovascular factors (total cholesterol, triglycerides, LDL-C); type 1/2 diabetes; testosterone, estrogen; osteoporosis, bone mineral density, serum 25-Hydroxyvitamin vitamin D and cervical spondylosis (Supplementary Tables S1–S3).

### 3.3 Influence of education on candidate mediating factors

Among them, education is a protective factor for smoking heaviness (β: 0.373; se: 0.032; *p* < 0.05; OR:0.69; [95%CI: 0.646–0.734]) and BFP (β: 0.290; se: 0.019; *p* < 0.05; OR:0.75; [95%CI: 0.720–0.777]). Besides, education is a risk factor for time spent using computer (β: 0.337; se: 0.016; *p* < 0.05; OR:1.40; [95%CI: 1.359–1.444]), time spent watching TV (β: 0.401; se: 0.013; *p* < 0.05; OR:0.67; [95%CI: 0.653–0.687]), BMI (β: 0.344; se: 0.026; *p* < 0.05; OR:0.71; [95%CI: 0.673–0.746]), HDL(β: 0.144; se: 0.034; *p* < 0.05; OR:1.15; [95%CI: 1.080–1.233]), major depression(β: 0.241; se: 0.036; *p* < 0.05; OR:0.79; [95%CI: 0.732–0.843]), SHBG(β: 0.109; se: 0.048; *p* < 0.05; OR:1.12; [95%CI: 1.016–1.225]) and sitting height (β: 0.150; se: 0.026; *p* < 0.05; OR:1.16; [95%CI: 1.103–1.222]) (Supplementary Table S4).

### 3.4 Influence of mediating factors on cervical spondylosis with adjustment for education

**FIGURE 3:**
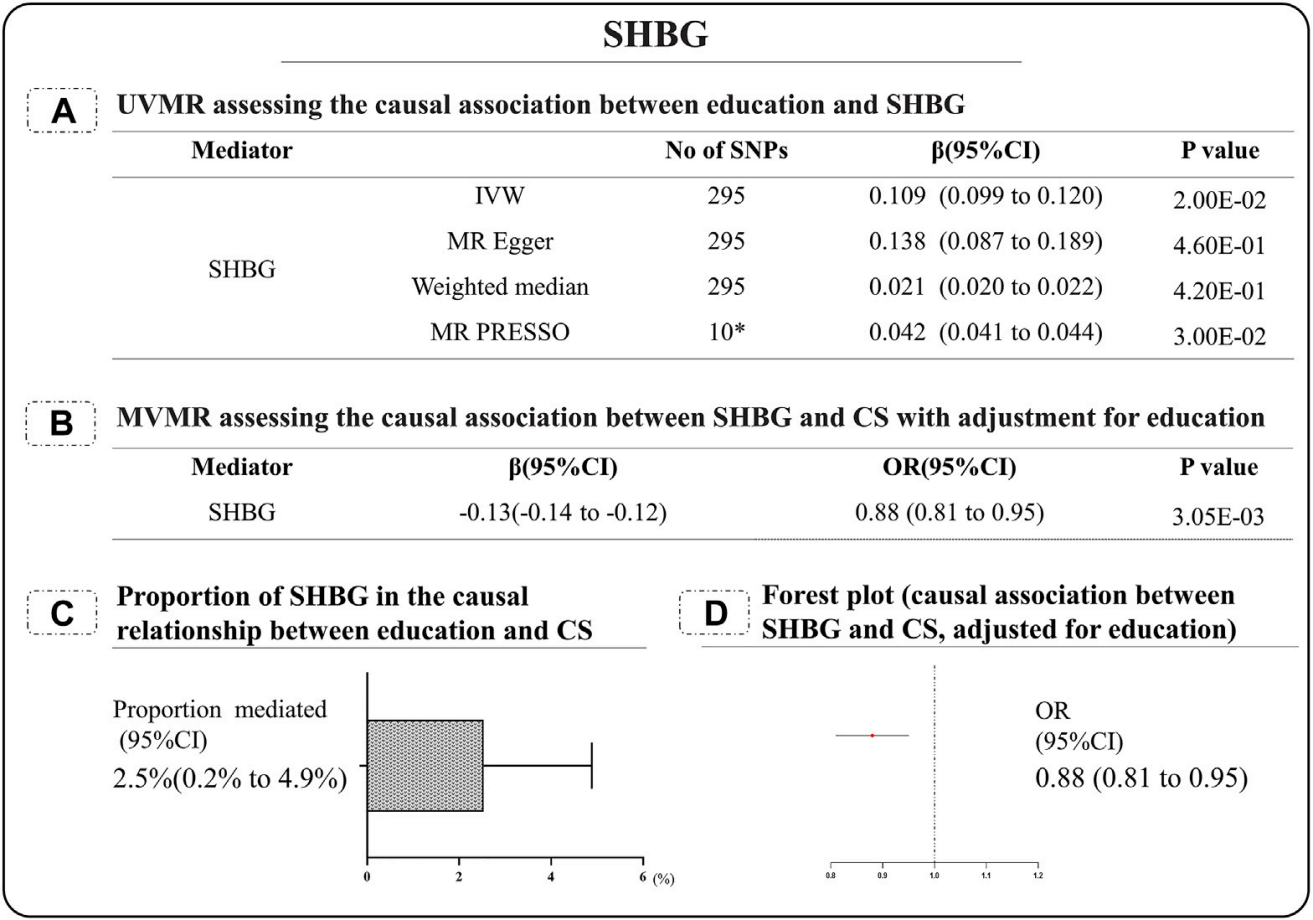
SHBG-related analysis results. **(A)** UVMR assessing the causal association between education and SHBG; **(B)** MVMR assessing the causal association between SHBG and CS with adjustment for education; **(C)** Proportion of SHBG in the causal relationship between education and CS; **(D)** Forest plot (causal association between SHBG and CS, adjusted for education). SHBG: Sex hormone binding globulin; CS cervical spondylosis.

It was estimated that the proportion of educational mediated effects on cervical spondylosis through SHBG as (2.5%; [95%CI:0.2%-4.9%]) (Figure 3).

## 4 Discussion

In this study, we assessed the causal association of cognitive, intellectual, educational, different lifestyles, health factors with cervical spondylosis by MR. Cognition, intelligence, and education have proven to be closely linked and inseparable (Savage et al., 2018; Wang et al., 2023). Studies have shown that higher educational attainment is a protective factor for cervical spondylosis (Sun et al., 2023). Factors such as smoking, obesity, exercise, lifestyle and cardiovascular disease may be related to the risk of cervical spondylosis (Theodore, 2020; Sun et al., 2023). At present, the mediating role of cardiovascular factors in education and cervical spondylosis has been demonstrated (Sun et al., 2023). However, the effects of different lifestyle and health factors on cervical spondylosis have not been elaborated by studies. This article will focus on the influence of different lifestyle and health factors on cervical spondylosis. In addition, this study expands on previous research by increasing the evidence on the total causal effect of cognitive function on cervical spondylosis, and for the first time identifies higher education as a protective factor for cervical spondylosis independent of intelligence and cognition, providing new insights into the possible pathogenesis of cervical spondylosis.

First, we conducted MR analysis of the association of cognition, intelligence, education with cervical spondylosis. The results pointed out that all three were causally related to the onset of cervical spondylosis. However, when the three are adjusted or any of the two, only education have an independent causal association with cervical spondylosis. Compared with the heritability of cognition and intelligence, educational attainment is more susceptible to other factors, which has lasting effects on people’s socioeconomic resources and status, as well as their lifestyle (Lawrence, 2017). In addition, educational attainment can be used as a proxy indicator of knowledge acquisition and health opportunities in later life. This suggests that preventive measures for cervical spondylosis can be taken by improving educational policies.

Another finding of this study was to identify and quantify the mediating role of different lifestyle and health factors in the association between education and cervical spondylosis. Through MR analysis, UVMR results showed a causal relationship between smoking, time spent using computer, time spent on TV, sitting height, BFP, major depression, BMI, HDL, SHBG and cervical spondylosis in 26 candidate mediators. The effect of smoking, BMI on cervical spondylosis is the same as that of SUN Y et al. (Sun et al., 2023). These results support the influence of lifestyle on the incidence of cervical spondylosis. Interestingly, the effect of watching TV on cervical spondylosis was different from that of computer using. The reason why watching TV is a risk factor for cervical spondylosis may be affected by the following factors. Firstly, the distance between the TV and the people is far, many people are basically in a state of poor posture when watching TV on the sofa or chair, and the bad posture of watching TV may last for a long time. Poor posture may be a factor that increases the risk of cervical spondylosis. Secondly, some people fall asleep while watching TV, whether lying on the sofa or sleeping in a chair, people are also in a bad posture. All these may increase the risk of cervical spondylosis. Different from television, the current stage of the computer monitor can be freely adjusted the height and angle of the monitor, people can adjust the position of the monitor according to the real-time situation when using the computer. This results in the user not being in a state of poor neck posture for a long time. In addition, with the increase of computer display screens, people need to move their necks frequently when using computers to ensure that they can get all the content on the monitor. This activity may be unconscious, but it also reduces the incidence of cervical spondylosis to a certain extent. The specific reasons for this phenomenon need to be confirmed by further research. Furthermore, factors such as lifestyle (leisure activities, long standing time and walking, amount of exercise, work intensity, drinking alcohol, coffee intake, household income), health factors (type 1/2 diabetes, osteoporosis, bone mineral density, serum vitamin D) were not found to have a clear causal relationship with cervical spondylosis. Promoting lifestyle changes through health education and developing good lifestyle habits may be used as a preventive measure for the risk of cervical spondylosis.

In further studies, when adjusted for education, only SHBG was still statistically significant in relation to cervical spondylosis. Education prevents the risk of cervical spondylosis by reducing SHBG. They mediated the protective effect of education by 2.5%. The discovery of SHBG is of great interest. SHBG is a protein produced by the liver that binds to and transports steroids such as estradiol and testosterone in plasma (Xing et al., 2022). Plasma concentrations of SHBG were positively correlated with estrogen and thyroid hormones, and negatively correlated with androgens (Selby, 1990). This may suggest to some extent that the effects of SHBG on education and cervical spondylosis are sex-related. However, we found that there was no causal relationship between androgens and cervical spondylosis, and the effect of estrogen on cervical spondylosis cannot be carried out because a suitable GWAS dataset has not been found, and further research is needed to explore the relationship. Excitingly, plasma concentrations of SHBG were shown to be associated with insulin concentrations and obesity (Xing et al., 2022). Our study found that there is no clear causal relationship between cervical spondylosis and type 1 and 2 diabetes. Studies have shown that SHBG is negatively correlated with BMI (Nokoff et al., 2019). After our MR analysis of SHBG, BMI and cervical spondylosis, we found that there was no clear causal relationship between SHBG and BMI, so BMI could not be used as an intermediary factor of SHBG for cervical spondylosis. It is a good research direction to further explore the relationship between SHBG and cervical spondylosis in follow-up research.

To ensure the accuracy of the results, we used the MVMR sensitivity analysis to support the IVW estimates and tried to ensure that each analysis accommodated different assumptions about genetic pleiotropy. In addition, we screen mediators through rigorous criteria to reduce reverse causality between intermediaries and education, thus ensuring accurate and reasonable results. However, our study still has some limitations. Although we focused on cervical spondylosis, whether this result is universal for different types and causes of cervical spondylosis still needs further discussion. Secondly, the study population had certain limitations, only for individuals of European ancestry, and the generalization of the results needs further research. In addition, although all the selected people are European, there are still regional differences between countries, suggesting that there may still be population heterogeneity in this study. Thirdly, the heterogeneity of SNPs can skew and affect the results. In future studies, we need more diverse populations, larger sample sizes and more diverse research methods to further validate this result.

## 5 Conclusion

In summary, this study elaborates the causal protective effect of education independent of intelligence and cognitive ability on cervical spondylosis, and outlines the causal mediating factors (SHBG) and factors that are causally associated with the onset of cervical spondylosis. This study further complements the causal evidence for the etiology of cervical spondylosis, and provides new ideas for prevention and intervention to control cervical spondylosis and its related disease burden.

## Data Availability

The original contributions presented in the study are included in the article/Supplementary Material, further inquiries can be directed to the corresponding author.
