# Supplementary material for: Causal associations of cognition, intelligence, education, health and lifestyle factors with cervical spondylosis: a mendelian randomization study
Source: Front Genet. 2024 Apr 25;15:1297213. doi: 10.3389/fgene.2024.1297213 (PMC11079178; doi:10.3389/fgene.2024.1297213)
Supplement: Supplementary file 1 [file DataSheet1.zip › Supplementary Table S1.pptx]

## Slide 1
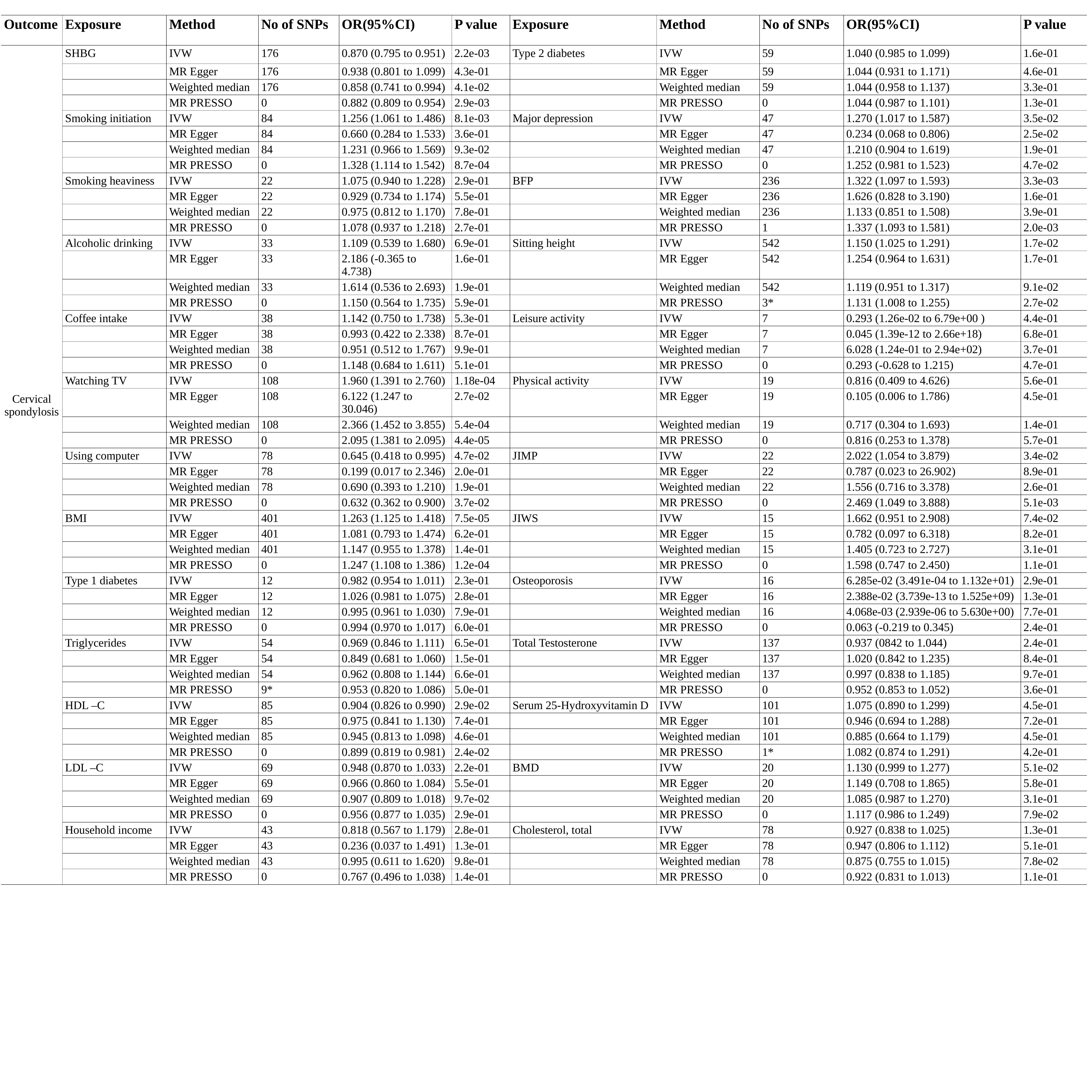

| Outcome | Exposure | Method | No of SNPs | OR(95%CI) | P value | Exposure | Method | No of SNPs | OR(95%CI) | P value |
| --- | --- | --- | --- | --- | --- | --- | --- | --- | --- | --- |
| Cervical spondylosis | SHBG | IVW | 176 | 0.870 (0.795 to 0.951) | 2.2e-03 | Type 2 diabetes | IVW | 59 | 1.040 (0.985 to 1.099) | 1.6e-01 |
| | | MR Egger | 176 | 0.938 (0.801 to 1.099) | 4.3e-01 | | MR Egger | 59 | 1.044 (0.931 to 1.171) | 4.6e-01 |
| | | Weighted median | 176 | 0.858 (0.741 to 0.994) | 4.1e-02 | | Weighted median | 59 | 1.044 (0.958 to 1.137) | 3.3e-01 |
| | | MR PRESSO | 0 | 0.882 (0.809 to 0.954) | 2.9e-03 | | MR PRESSO | 0 | 1.044 (0.987 to 1.101) | 1.3e-01 |
| | Smoking initiation | IVW | 84 | 1.256 (1.061 to 1.486) | 8.1e-03 | Major depression | IVW | 47 | 1.270 (1.017 to 1.587) | 3.5e-02 |
| | | MR Egger | 84 | 0.660 (0.284 to 1.533) | 3.6e-01 | | MR Egger | 47 | 0.234 (0.068 to 0.806) | 2.5e-02 |
| | | Weighted median | 84 | 1.231 (0.966 to 1.569) | 9.3e-02 | | Weighted median | 47 | 1.210 (0.904 to 1.619) | 1.9e-01 |
| | | MR PRESSO | 0 | 1.328 (1.114 to 1.542) | 8.7e-04 | | MR PRESSO | 0 | 1.252 (0.981 to 1.523) | 4.7e-02 |
| | Smoking heaviness | IVW | 22 | 1.075 (0.940 to 1.228) | 2.9e-01 | BFP | IVW | 236 | 1.322 (1.097 to 1.593) | 3.3e-03 |
| | | MR Egger | 22 | 0.929 (0.734 to 1.174) | 5.5e-01 | | MR Egger | 236 | 1.626 (0.828 to 3.190) | 1.6e-01 |
| | | Weighted median | 22 | 0.975 (0.812 to 1.170) | 7.8e-01 | | Weighted median | 236 | 1.133 (0.851 to 1.508) | 3.9e-01 |
| | | MR PRESSO | 0 | 1.078 (0.937 to 1.218) | 2.7e-01 | | MR PRESSO | 1 | 1.337 (1.093 to 1.581) | 2.0e-03 |
| | Alcoholic drinking | IVW | 33 | 1.109 (0.539 to 1.680) | 6.9e-01 | Sitting height | IVW | 542 | 1.150 (1.025 to 1.291) | 1.7e-02 |
| | | MR Egger | 33 | 2.186 (-0.365 to 4.738) | 1.6e-01 | | MR Egger | 542 | 1.254 (0.964 to 1.631) | 1.7e-01 |
| | | Weighted median | 33 | 1.614 (0.536 to 2.693) | 1.9e-01 | | Weighted median | 542 | 1.119 (0.951 to 1.317) | 9.1e-02 |
| | | MR PRESSO | 0 | 1.150 (0.564 to 1.735) | 5.9e-01 | | MR PRESSO | 3\* | 1.131 (1.008 to 1.255) | 2.7e-02 |
| | Coffee intake | IVW | 38 | 1.142 (0.750 to 1.738) | 5.3e-01 | Leisure activity | IVW | 7 | 0.293 (1.26e-02 to 6.79e+00 ) | 4.4e-01 |
| | | MR Egger | 38 | 0.993 (0.422 to 2.338) | 8.7e-01 | | MR Egger | 7 | 0.045 (1.39e-12 to 2.66e+18) | 6.8e-01 |
| | | Weighted median | 38 | 0.951 (0.512 to 1.767) | 9.9e-01 | | Weighted median | 7 | 6.028 (1.24e-01 to 2.94e+02) | 3.7e-01 |
| | | MR PRESSO | 0 | 1.148 (0.684 to 1.611) | 5.1e-01 | | MR PRESSO | 0 | 0.293 (-0.628 to 1.215) | 4.7e-01 |
| | Watching TV | IVW | 108 | 1.960 (1.391 to 2.760) | 1.18e-04 | Physical activity | IVW | 19 | 0.816 (0.409 to 4.626) | 5.6e-01 |
| | | MR Egger | 108 | 6.122 (1.247 to 30.046) | 2.7e-02 | | MR Egger | 19 | 0.105 (0.006 to 1.786) | 4.5e-01 |
| | | Weighted median | 108 | 2.366 (1.452 to 3.855) | 5.4e-04 | | Weighted median | 19 | 0.717 (0.304 to 1.693) | 1.4e-01 |
| | | MR PRESSO | 0 | 2.095 (1.381 to 2.095) | 4.4e-05 | | MR PRESSO | 0 | 0.816 (0.253 to 1.378) | 5.7e-01 |
| | Using computer | IVW | 78 | 0.645 (0.418 to 0.995) | 4.7e-02 | JIMP | IVW | 22 | 2.022 (1.054 to 3.879) | 3.4e-02 |
| | | MR Egger | 78 | 0.199 (0.017 to 2.346) | 2.0e-01 | | MR Egger | 22 | 0.787 (0.023 to 26.902) | 8.9e-01 |
| | | Weighted median | 78 | 0.690 (0.393 to 1.210) | 1.9e-01 | | Weighted median | 22 | 1.556 (0.716 to 3.378) | 2.6e-01 |
| | | MR PRESSO | 0 | 0.632 (0.362 to 0.900) | 3.7e-02 | | MR PRESSO | 0 | 2.469 (1.049 to 3.888) | 5.1e-03 |
| | BMI | IVW | 401 | 1.263 (1.125 to 1.418) | 7.5e-05 | JIWS | IVW | 15 | 1.662 (0.951 to 2.908) | 7.4e-02 |
| | | MR Egger | 401 | 1.081 (0.793 to 1.474) | 6.2e-01 | | MR Egger | 15 | 0.782 (0.097 to 6.318) | 8.2e-01 |
| | | Weighted median | 401 | 1.147 (0.955 to 1.378) | 1.4e-01 | | Weighted median | 15 | 1.405 (0.723 to 2.727) | 3.1e-01 |
| | | MR PRESSO | 0 | 1.247 (1.108 to 1.386) | 1.2e-04 | | MR PRESSO | 0 | 1.598 (0.747 to 2.450) | 1.1e-01 |
| | Type 1 diabetes | IVW | 12 | 0.982 (0.954 to 1.011) | 2.3e-01 | Osteoporosis | IVW | 16 | 6.285e-02 (3.491e-04 to 1.132e+01) | 2.9e-01 |
| | | MR Egger | 12 | 1.026 (0.981 to 1.075) | 2.8e-01 | | MR Egger | 16 | 2.388e-02 (3.739e-13 to 1.525e+09) | 1.3e-01 |
| | | Weighted median | 12 | 0.995 (0.961 to 1.030) | 7.9e-01 | | Weighted median | 16 | 4.068e-03 (2.939e-06 to 5.630e+00) | 7.7e-01 |
| | | MR PRESSO | 0 | 0.994 (0.970 to 1.017) | 6.0e-01 | | MR PRESSO | 0 | 0.063 (-0.219 to 0.345) | 2.4e-01 |
| | Triglycerides | IVW | 54 | 0.969 (0.846 to 1.111) | 6.5e-01 | Total Testosterone | IVW | 137 | 0.937 (0842 to 1.044) | 2.4e-01 |
| | | MR Egger | 54 | 0.849 (0.681 to 1.060) | 1.5e-01 | | MR Egger | 137 | 1.020 (0.842 to 1.235) | 8.4e-01 |
| | | Weighted median | 54 | 0.962 (0.808 to 1.144) | 6.6e-01 | | Weighted median | 137 | 0.997 (0.838 to 1.185) | 9.7e-01 |
| | | MR PRESSO | 9\* | 0.953 (0.820 to 1.086) | 5.0e-01 | | MR PRESSO | 0 | 0.952 (0.853 to 1.052) | 3.6e-01 |
| | HDL –C | IVW | 85 | 0.904 (0.826 to 0.990) | 2.9e-02 | Serum 25-Hydroxyvitamin D | IVW | 101 | 1.075 (0.890 to 1.299) | 4.5e-01 |
| | | MR Egger | 85 | 0.975 (0.841 to 1.130) | 7.4e-01 | | MR Egger | 101 | 0.946 (0.694 to 1.288) | 7.2e-01 |
| | | Weighted median | 85 | 0.945 (0.813 to 1.098) | 4.6e-01 | | Weighted median | 101 | 0.885 (0.664 to 1.179) | 4.5e-01 |
| | | MR PRESSO | 0 | 0.899 (0.819 to 0.981) | 2.4e-02 | | MR PRESSO | 1\* | 1.082 (0.874 to 1.291) | 4.2e-01 |
| | LDL –C | IVW | 69 | 0.948 (0.870 to 1.033) | 2.2e-01 | BMD | IVW | 20 | 1.130 (0.999 to 1.277) | 5.1e-02 |
| | | MR Egger | 69 | 0.966 (0.860 to 1.084) | 5.5e-01 | | MR Egger | 20 | 1.149 (0.708 to 1.865) | 5.8e-01 |
| | | Weighted median | 69 | 0.907 (0.809 to 1.018) | 9.7e-02 | | Weighted median | 20 | 1.085 (0.987 to 1.270) | 3.1e-01 |
| | | MR PRESSO | 0 | 0.956 (0.877 to 1.035) | 2.9e-01 | | MR PRESSO | 0 | 1.117 (0.986 to 1.249) | 7.9e-02 |
| | Household income | IVW | 43 | 0.818 (0.567 to 1.179) | 2.8e-01 | Cholesterol, total | IVW | 78 | 0.927 (0.838 to 1.025) | 1.3e-01 |
| | | MR Egger | 43 | 0.236 (0.037 to 1.491) | 1.3e-01 | | MR Egger | 78 | 0.947 (0.806 to 1.112) | 5.1e-01 |
| | | Weighted median | 43 | 0.995 (0.611 to 1.620) | 9.8e-01 | | Weighted median | 78 | 0.875 (0.755 to 1.015) | 7.8e-02 |
| | | MR PRESSO | 0 | 0.767 (0.496 to 1.038) | 1.4e-01 | | MR PRESSO | 0 | 0.922 (0.831 to 1.013) | 1.1e-01 |
