# Supplementary material for: Causal associations of cognition, intelligence, education, health and lifestyle factors with cervical spondylosis: a mendelian randomization study
Source: Front Genet. 2024 Apr 25;15:1297213. doi: 10.3389/fgene.2024.1297213 (PMC11079178; doi:10.3389/fgene.2024.1297213)
Supplement: Supplementary file 1 [file DataSheet1.zip › Supplementary Table S2.pptx]

## Slide 1
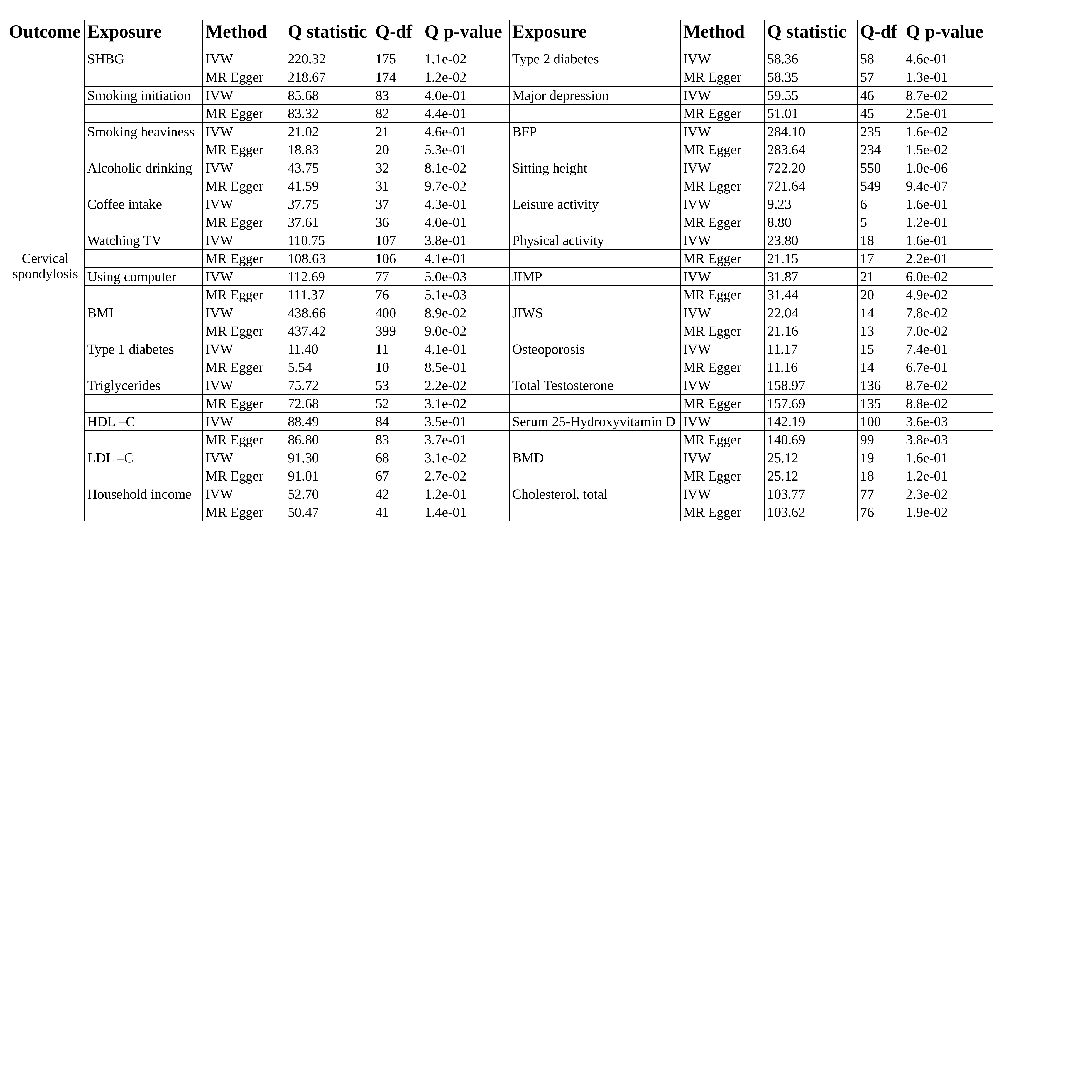

| Outcome | Exposure | Method | Q statistic | Q-df | Q p-value | Exposure | Method | Q statistic | Q-df | Q p-value |
| --- | --- | --- | --- | --- | --- | --- | --- | --- | --- | --- |
| Cervical spondylosis | SHBG | IVW | 220.32 | 175 | 1.1e-02 | Type 2 diabetes | IVW | 58.36 | 58 | 4.6e-01 |
| | | MR Egger | 218.67 | 174 | 1.2e-02 | | MR Egger | 58.35 | 57 | 1.3e-01 |
| | Smoking initiation | IVW | 85.68 | 83 | 4.0e-01 | Major depression | IVW | 59.55 | 46 | 8.7e-02 |
| | | MR Egger | 83.32 | 82 | 4.4e-01 | | MR Egger | 51.01 | 45 | 2.5e-01 |
| | Smoking heaviness | IVW | 21.02 | 21 | 4.6e-01 | BFP | IVW | 284.10 | 235 | 1.6e-02 |
| | | MR Egger | 18.83 | 20 | 5.3e-01 | | MR Egger | 283.64 | 234 | 1.5e-02 |
| | Alcoholic drinking | IVW | 43.75 | 32 | 8.1e-02 | Sitting height | IVW | 722.20 | 550 | 1.0e-06 |
| | | MR Egger | 41.59 | 31 | 9.7e-02 | | MR Egger | 721.64 | 549 | 9.4e-07 |
| | Coffee intake | IVW | 37.75 | 37 | 4.3e-01 | Leisure activity | IVW | 9.23 | 6 | 1.6e-01 |
| | | MR Egger | 37.61 | 36 | 4.0e-01 | | MR Egger | 8.80 | 5 | 1.2e-01 |
| | Watching TV | IVW | 110.75 | 107 | 3.8e-01 | Physical activity | IVW | 23.80 | 18 | 1.6e-01 |
| | | MR Egger | 108.63 | 106 | 4.1e-01 | | MR Egger | 21.15 | 17 | 2.2e-01 |
| | Using computer | IVW | 112.69 | 77 | 5.0e-03 | JIMP | IVW | 31.87 | 21 | 6.0e-02 |
| | | MR Egger | 111.37 | 76 | 5.1e-03 | | MR Egger | 31.44 | 20 | 4.9e-02 |
| | BMI | IVW | 438.66 | 400 | 8.9e-02 | JIWS | IVW | 22.04 | 14 | 7.8e-02 |
| | | MR Egger | 437.42 | 399 | 9.0e-02 | | MR Egger | 21.16 | 13 | 7.0e-02 |
| | Type 1 diabetes | IVW | 11.40 | 11 | 4.1e-01 | Osteoporosis | IVW | 11.17 | 15 | 7.4e-01 |
| | | MR Egger | 5.54 | 10 | 8.5e-01 | | MR Egger | 11.16 | 14 | 6.7e-01 |
| | Triglycerides | IVW | 75.72 | 53 | 2.2e-02 | Total Testosterone | IVW | 158.97 | 136 | 8.7e-02 |
| | | MR Egger | 72.68 | 52 | 3.1e-02 | | MR Egger | 157.69 | 135 | 8.8e-02 |
| | HDL –C | IVW | 88.49 | 84 | 3.5e-01 | Serum 25-Hydroxyvitamin D | IVW | 142.19 | 100 | 3.6e-03 |
| | | MR Egger | 86.80 | 83 | 3.7e-01 | | MR Egger | 140.69 | 99 | 3.8e-03 |
| | LDL –C | IVW | 91.30 | 68 | 3.1e-02 | BMD | IVW | 25.12 | 19 | 1.6e-01 |
| | | MR Egger | 91.01 | 67 | 2.7e-02 | | MR Egger | 25.12 | 18 | 1.2e-01 |
| | Household income | IVW | 52.70 | 42 | 1.2e-01 | Cholesterol, total | IVW | 103.77 | 77 | 2.3e-02 |
| | | MR Egger | 50.47 | 41 | 1.4e-01 | | MR Egger | 103.62 | 76 | 1.9e-02 |
