# Supplementary material for: Causal associations of cognition, intelligence, education, health and lifestyle factors with cervical spondylosis: a mendelian randomization study
Source: Front Genet. 2024 Apr 25;15:1297213. doi: 10.3389/fgene.2024.1297213 (PMC11079178; doi:10.3389/fgene.2024.1297213)
Supplement: Supplementary file 1 [file DataSheet1.zip › Supplementary Table S3.pptx]

## Slide 1
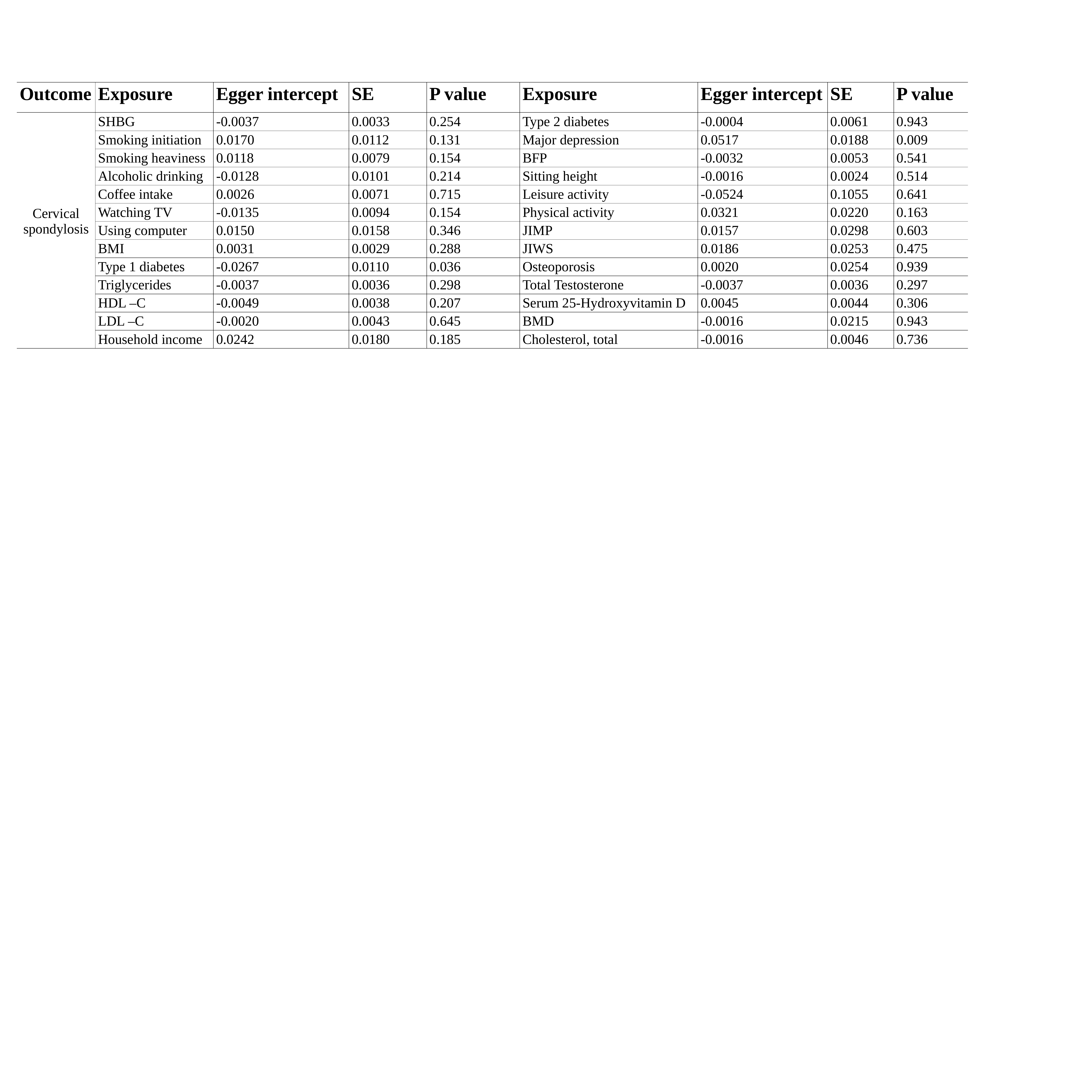

| Outcome | Exposure | Egger intercept | SE | P value | Exposure | Egger intercept | SE | P value |
| --- | --- | --- | --- | --- | --- | --- | --- | --- |
| Cervical spondylosis | SHBG | -0.0037 | 0.0033 | 0.254 | Type 2 diabetes | -0.0004 | 0.0061 | 0.943 |
| | Smoking initiation | 0.0170 | 0.0112 | 0.131 | Major depression | 0.0517 | 0.0188 | 0.009 |
| | Smoking heaviness | 0.0118 | 0.0079 | 0.154 | BFP | -0.0032 | 0.0053 | 0.541 |
| | Alcoholic drinking | -0.0128 | 0.0101 | 0.214 | Sitting height | -0.0016 | 0.0024 | 0.514 |
| | Coffee intake | 0.0026 | 0.0071 | 0.715 | Leisure activity | -0.0524 | 0.1055 | 0.641 |
| | Watching TV | -0.0135 | 0.0094 | 0.154 | Physical activity | 0.0321 | 0.0220 | 0.163 |
| | Using computer | 0.0150 | 0.0158 | 0.346 | JIMP | 0.0157 | 0.0298 | 0.603 |
| | BMI | 0.0031 | 0.0029 | 0.288 | JIWS | 0.0186 | 0.0253 | 0.475 |
| | Type 1 diabetes | -0.0267 | 0.0110 | 0.036 | Osteoporosis | 0.0020 | 0.0254 | 0.939 |
| | Triglycerides | -0.0037 | 0.0036 | 0.298 | Total Testosterone | -0.0037 | 0.0036 | 0.297 |
| | HDL –C | -0.0049 | 0.0038 | 0.207 | Serum 25-Hydroxyvitamin D | 0.0045 | 0.0044 | 0.306 |
| | LDL –C | -0.0020 | 0.0043 | 0.645 | BMD | -0.0016 | 0.0215 | 0.943 |
| | Household income | 0.0242 | 0.0180 | 0.185 | Cholesterol, total | -0.0016 | 0.0046 | 0.736 |
