# Supplementary material for: Causal associations of cognition, intelligence, education, health and lifestyle factors with cervical spondylosis: a mendelian randomization study
Source: Front Genet. 2024 Apr 25;15:1297213. doi: 10.3389/fgene.2024.1297213 (PMC11079178; doi:10.3389/fgene.2024.1297213)
Supplement: Supplementary file 1 [file DataSheet1.zip › Supplementary Table S4.pptx]

## Slide 1
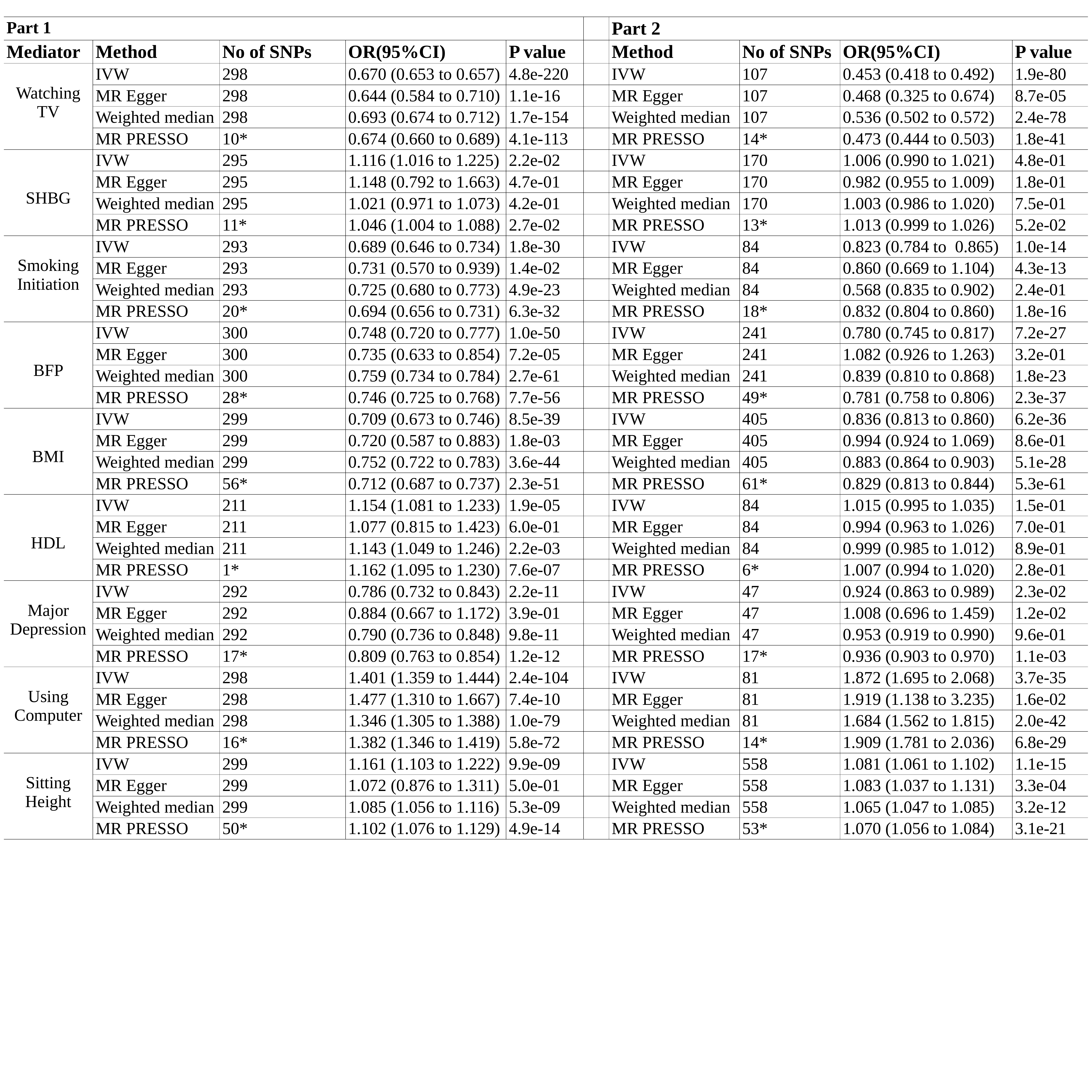

| Part 1 | | | | | | Part 2 | | | |
| --- | --- | --- | --- | --- | --- | --- | --- | --- | --- |
| Mediator | Method | No of SNPs | OR(95%CI) | P value | | Method | No of SNPs | OR(95%CI) | P value |
| Watching TV | IVW | 298 | 0.670 (0.653 to 0.657) | 4.8e-220 | | IVW | 107 | 0.453 (0.418 to 0.492) | 1.9e-80 |
| | MR Egger | 298 | 0.644 (0.584 to 0.710) | 1.1e-16 | | MR Egger | 107 | 0.468 (0.325 to 0.674) | 8.7e-05 |
| | Weighted median | 298 | 0.693 (0.674 to 0.712) | 1.7e-154 | | Weighted median | 107 | 0.536 (0.502 to 0.572) | 2.4e-78 |
| | MR PRESSO | 10\* | 0.674 (0.660 to 0.689) | 4.1e-113 | | MR PRESSO | 14\* | 0.473 (0.444 to 0.503) | 1.8e-41 |
| SHBG | IVW | 295 | 1.116 (1.016 to 1.225) | 2.2e-02 | | IVW | 170 | 1.006 (0.990 to 1.021) | 4.8e-01 |
| | MR Egger | 295 | 1.148 (0.792 to 1.663) | 4.7e-01 | | MR Egger | 170 | 0.982 (0.955 to 1.009) | 1.8e-01 |
| | Weighted median | 295 | 1.021 (0.971 to 1.073) | 4.2e-01 | | Weighted median | 170 | 1.003 (0.986 to 1.020) | 7.5e-01 |
| | MR PRESSO | 11\* | 1.046 (1.004 to 1.088) | 2.7e-02 | | MR PRESSO | 13\* | 1.013 (0.999 to 1.026) | 5.2e-02 |
| Smoking Initiation | IVW | 293 | 0.689 (0.646 to 0.734) | 1.8e-30 | | IVW | 84 | 0.823 (0.784 to 0.865) | 1.0e-14 |
| | MR Egger | 293 | 0.731 (0.570 to 0.939) | 1.4e-02 | | MR Egger | 84 | 0.860 (0.669 to 1.104) | 4.3e-13 |
| | Weighted median | 293 | 0.725 (0.680 to 0.773) | 4.9e-23 | | Weighted median | 84 | 0.568 (0.835 to 0.902) | 2.4e-01 |
| | MR PRESSO | 20\* | 0.694 (0.656 to 0.731) | 6.3e-32 | | MR PRESSO | 18\* | 0.832 (0.804 to 0.860) | 1.8e-16 |
| BFP | IVW | 300 | 0.748 (0.720 to 0.777) | 1.0e-50 | | IVW | 241 | 0.780 (0.745 to 0.817) | 7.2e-27 |
| | MR Egger | 300 | 0.735 (0.633 to 0.854) | 7.2e-05 | | MR Egger | 241 | 1.082 (0.926 to 1.263) | 3.2e-01 |
| | Weighted median | 300 | 0.759 (0.734 to 0.784) | 2.7e-61 | | Weighted median | 241 | 0.839 (0.810 to 0.868) | 1.8e-23 |
| | MR PRESSO | 28\* | 0.746 (0.725 to 0.768) | 7.7e-56 | | MR PRESSO | 49\* | 0.781 (0.758 to 0.806) | 2.3e-37 |
| BMI | IVW | 299 | 0.709 (0.673 to 0.746) | 8.5e-39 | | IVW | 405 | 0.836 (0.813 to 0.860) | 6.2e-36 |
| | MR Egger | 299 | 0.720 (0.587 to 0.883) | 1.8e-03 | | MR Egger | 405 | 0.994 (0.924 to 1.069) | 8.6e-01 |
| | Weighted median | 299 | 0.752 (0.722 to 0.783) | 3.6e-44 | | Weighted median | 405 | 0.883 (0.864 to 0.903) | 5.1e-28 |
| | MR PRESSO | 56\* | 0.712 (0.687 to 0.737) | 2.3e-51 | | MR PRESSO | 61\* | 0.829 (0.813 to 0.844) | 5.3e-61 |
| HDL | IVW | 211 | 1.154 (1.081 to 1.233) | 1.9e-05 | | IVW | 84 | 1.015 (0.995 to 1.035) | 1.5e-01 |
| | MR Egger | 211 | 1.077 (0.815 to 1.423) | 6.0e-01 | | MR Egger | 84 | 0.994 (0.963 to 1.026) | 7.0e-01 |
| | Weighted median | 211 | 1.143 (1.049 to 1.246) | 2.2e-03 | | Weighted median | 84 | 0.999 (0.985 to 1.012) | 8.9e-01 |
| | MR PRESSO | 1\* | 1.162 (1.095 to 1.230) | 7.6e-07 | | MR PRESSO | 6\* | 1.007 (0.994 to 1.020) | 2.8e-01 |
| Major Depression | IVW | 292 | 0.786 (0.732 to 0.843) | 2.2e-11 | | IVW | 47 | 0.924 (0.863 to 0.989) | 2.3e-02 |
| | MR Egger | 292 | 0.884 (0.667 to 1.172) | 3.9e-01 | | MR Egger | 47 | 1.008 (0.696 to 1.459) | 1.2e-02 |
| | Weighted median | 292 | 0.790 (0.736 to 0.848) | 9.8e-11 | | Weighted median | 47 | 0.953 (0.919 to 0.990) | 9.6e-01 |
| | MR PRESSO | 17\* | 0.809 (0.763 to 0.854) | 1.2e-12 | | MR PRESSO | 17\* | 0.936 (0.903 to 0.970) | 1.1e-03 |
| Using Computer | IVW | 298 | 1.401 (1.359 to 1.444) | 2.4e-104 | | IVW | 81 | 1.872 (1.695 to 2.068) | 3.7e-35 |
| | MR Egger | 298 | 1.477 (1.310 to 1.667) | 7.4e-10 | | MR Egger | 81 | 1.919 (1.138 to 3.235) | 1.6e-02 |
| | Weighted median | 298 | 1.346 (1.305 to 1.388) | 1.0e-79 | | Weighted median | 81 | 1.684 (1.562 to 1.815) | 2.0e-42 |
| | MR PRESSO | 16\* | 1.382 (1.346 to 1.419) | 5.8e-72 | | MR PRESSO | 14\* | 1.909 (1.781 to 2.036) | 6.8e-29 |
| Sitting Height | IVW | 299 | 1.161 (1.103 to 1.222) | 9.9e-09 | | IVW | 558 | 1.081 (1.061 to 1.102) | 1.1e-15 |
| | MR Egger | 299 | 1.072 (0.876 to 1.311) | 5.0e-01 | | MR Egger | 558 | 1.083 (1.037 to 1.131) | 3.3e-04 |
| | Weighted median | 299 | 1.085 (1.056 to 1.116) | 5.3e-09 | | Weighted median | 558 | 1.065 (1.047 to 1.085) | 3.2e-12 |
| | MR PRESSO | 50\* | 1.102 (1.076 to 1.129) | 4.9e-14 | | MR PRESSO | 53\* | 1.070 (1.056 to 1.084) | 3.1e-21 |
