# Supplementary material for: Causal associations of cognition, intelligence, education, health and lifestyle factors with cervical spondylosis: a mendelian randomization study
Source: Front Genet. 2024 Apr 25;15:1297213. doi: 10.3389/fgene.2024.1297213 (PMC11079178; doi:10.3389/fgene.2024.1297213)
Supplement: Supplementary file 1 [file DataSheet1.zip › Supplementary Table S5.pptx]

## Slide 1
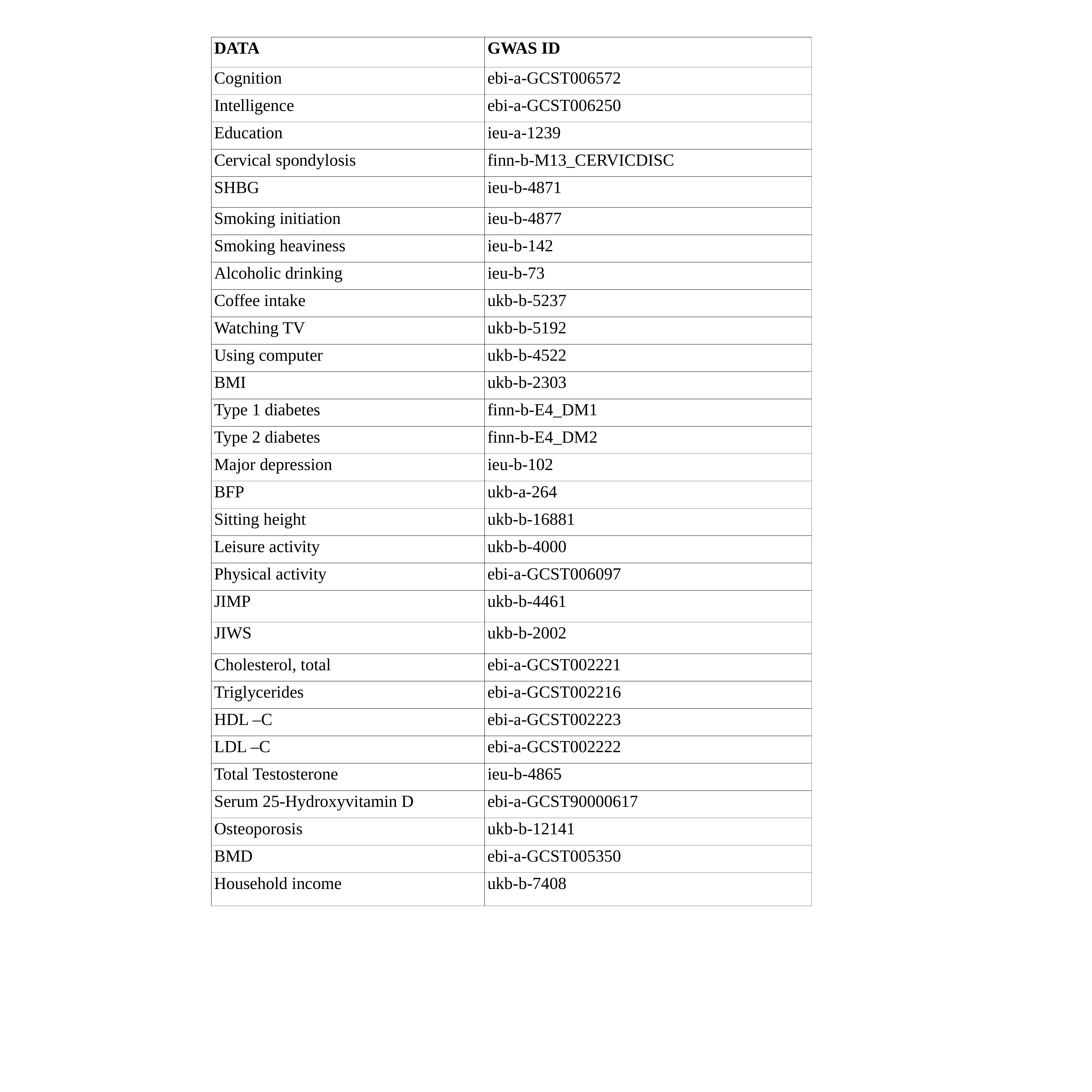

| DATA | GWAS ID |
| --- | --- |
| Cognition | ebi-a-GCST006572 |
| Intelligence | ebi-a-GCST006250 |
| Education | ieu-a-1239 |
| Cervical spondylosis | finn-b-M13\_CERVICDISC |
| SHBG | ieu-b-4871 |
| Smoking initiation | ieu-b-4877 |
| Smoking heaviness | ieu-b-142 |
| Alcoholic drinking | ieu-b-73 |
| Coffee intake | ukb-b-5237 |
| Watching TV | ukb-b-5192 |
| Using computer | ukb-b-4522 |
| BMI | ukb-b-2303 |
| Type 1 diabetes | finn-b-E4\_DM1 |
| Type 2 diabetes | finn-b-E4\_DM2 |
| Major depression | ieu-b-102 |
| BFP | ukb-a-264 |
| Sitting height | ukb-b-16881 |
| Leisure activity | ukb-b-4000 |
| Physical activity | ebi-a-GCST006097 |
| JIMP | ukb-b-4461 |
| JIWS | ukb-b-2002 |
| Cholesterol, total | ebi-a-GCST002221 |
| Triglycerides | ebi-a-GCST002216 |
| HDL –C | ebi-a-GCST002223 |
| LDL –C | ebi-a-GCST002222 |
| Total Testosterone | ieu-b-4865 |
| Serum 25-Hydroxyvitamin D | ebi-a-GCST90000617 |
| Osteoporosis | ukb-b-12141 |
| BMD | ebi-a-GCST005350 |
| Household income | ukb-b-7408 |
